# Myoinositol as a Safe and Alternative Approach in the Treatment of Infertile PCOS Women: A German Observational Study

**DOI:** 10.1155/2016/9537632

**Published:** 2016-08-23

**Authors:** Pedro-Antonio Regidor, Adolf Eduard Schindler

**Affiliations:** ^1^Frauenklinik München West, Schmiedwegerl 2-6, 81242 Munich, Germany; ^2^Institut für Medizinische Forschung, Hufelandstraße 55, 45122 Essen, Germany

## Abstract

The use of 2 × 2000 mg myoinositol + 2 × 200 *μ*g folic acid per day is a safe and promising tool in the effective improvement of symptoms and infertility for patients with a polycystic ovary syndrome (PCOS). Using a questionnaire an observational study was performed under German gynecologists to collect data on ovulation and pregnancy rates in PCOS patients with infertility. In this observational study, 3602 infertile women used myoinositol and folic acid between 2 and 3 months in a dosage of 2 × 2000 mg myoinositol + 2 × 200 *μ*g folic acid per day. In a subgroup of 32 patients, hormonal values for testosterone, free testosterone, and progesterone were analyzed before and after 12 weeks of treatment. The mean time of use was 10.2 weeks. During this time 70% of these women had a restored ovulation, and 545 pregnancies were obtained. This means a pregnancy rate of 15.1% of all the myoinositol and folic acid users. In 19 cases a concomitant medication with clomiphene or dexamethasone was used. One twin pregnancy was documented. Testosterone levels changed from 96.6 ng/ml to 43.3 ng/ml and progesterone from 2.1 ng/ml to 12.3 ng/ml (*p* < 0.05) after 12 weeks of treatment. No relevant side effects were present among the patients. This study could show that a new treatment option for patients with a PCOS and infertility is available. The achieved pregnancy rates are at least in an equivalent or even superior range than those reported by the use of metformin.

## 1. Introduction 

The PCOS is the most common cause of menstrual disorders, ovarian dysfunction, and infertility of women. Observational studies postulate that up to 15% of women suffer under this condition during their reproductive life. PCOS etiopathology is not clear, but most probably a strong genetic cause that is influenced by gestational environment and lifestyle seems to be the key factor. The most common features of PCOS are hyperandrogenism, chronic anovulation, typical PCOS ultrasound images, and skin issues such as acne, hirsutism, and seborrhea. Furthermore, recently it has been found that insulin resistance plays a key role in the clinical development of PCOS in almost all the women. Severe disorders of the insulin sensitivity with a compensatory hyperinsulinemic state not only in obese PCOS patients but also in lean women have been described, so that the hypothesis is strongly supported that the insulin resistance is independent of the weight [1]. In particular, the related hyperinsulinemia could induce an excess of androgens production in PCOS women through two different ways: first one is direct stimulation of ovaries to produce androgens, and the other one is the reduction of sex hormone binding globulin (SHBG) serum levels [2].

Due to the key role of insulin in the syndrome etiopathology, for many years, insulin sensitizers such as metformin, pioglitazone, or troglitazone have been considered as possible therapeutic options in the management of these problems. Metformin has been used in the last time on patients with a hyperinsulinemic status for the improvement of ovarian dysfunction with consecutive anovulation, irregular menstrual cycles, and infertility problems [3, 4]. Nevertheless metformin, when used in the therapeutic dose range, was shown to have several side effects such as flatulence, diarrhea, and nausea, so that many patients are unable to use this treatment option in gynecology for a longer period of time [5, 6].

Therefore, in parallel to the common use of metformin and other insulin sensitizer agents for the treatment of PCOS, in the recent years, other therapeutic alternatives have been investigated.

Myoinositol is one of the most interesting molecules that have been studied for the treatment of PCOS.

The substance inositol is a* chemical compound* with the formula C_6_H_12_O_6_ or (–CHOH–)_6_, a sixfold* alcohol* (*polyol*) of* cyclohexane*, with five equatorial and one axial hydroxyl group. It is widely found in nature. There exist nine different stereoisomer forms, but myoinositol is the most common one found in nature. In fact, myoinositol is very often found in many plants and in tissues of animals. The second most common form is D-chiro-inositol. It is important to distinguish between the lecithin formulation that is bioavailable for the human and the phytate formulation of corns that are not bioavailable. Foods with the highest concentration of myoinositol are fruits, beans, corns, and nuts [7].

Inositol was defined in the past as “myometrial sugar,” but it is indeed not a substance belonging to the carbohydrate group if we use modern definitions. Defining inositol as a vitamin B is also being discussed with controversy as inositol is not an essential substance and it can be produced in human cells from glucose [8]. In fact, several studies have proved that the inositol molecule is directly involved in the insulin cellular signaling.

**Figure 1:**
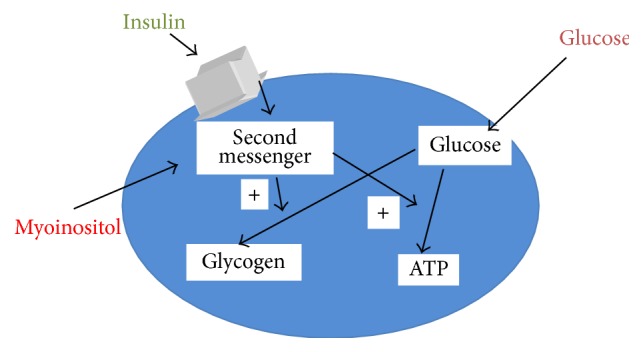
Mechanism of action of myoinositol in the cell.

Regarding PCOS, several studies have shown that one of the mechanisms of insulin deficiency has its rise from the inositolphosphoglycan (IPG) mediator and that a deficiency of inositol in the inositolphosphoglycans is responsible for insulin resistance. It has demonstrated that the administration of D-chiro-inositol (intracellularly converted from myoinositol) could reduce the insulin resistance [9] (see Figure 1).

Indeed, myoinositol, as a second messenger, plays an essential role for the signal pathways of cells. In particular, the action of myoinositol in a PCOS pathway would be related to an improved insulin sensitivity and a sequent increased intracellular glucose uptake [2, 10].

All these pieces of evidence have opened a new clinical interest on myoinositol, as a potential insulin sensitizer agent to be used as safe and effective option in PCOS patients, through the restoration of their metabolic profile and a consequent ovulation induction in infertile PCOS patients. Studies report also a very good safety profile of the molecule, even when administered up to 12 grams/day, where only mild gastrointestinal side effects have been reported [11].

The aim of this study was to determine the pregnancy rates under the use of a combination of myoinositol and folic acid in patients with a PCOS in Germany, to establish if this molecule can be used as a safer treatment option for the fertility improvement of this disease.

## 2. Patients and Methods 

A standardized questionnaire was created and a questionnaire (see Appendix) was presented to 245 gynecologists present in Germany, between June 2014 and March 2015. During this time reports were generated of 3602 women with a PCOS and infertility according to the Rotterdam classification. The women started with the intake of myoinositol and folic acid at a dosage of 2 × 2000 mg myoinositol and 2 × 200 *μ*g folic acid per day and used it for at least 2-3 months. The primary outcome of the study was to determine the ovulatory function restoration and the pregnancy rate after treatment. The pregnancies were documented by the gynecologists and registered in a database, and these women were followed up during the whole pregnancy. Secondary outcome was the evaluation of side effects reported in those patients undergoing treatment. In a subgroup of patients, hormonal values were also evaluated. The values investigated were testosterone, free testosterone, and progesterone. In this group of patients the pregnancies outcome has also been checked.

## 3. Results

The data of 3602 patients with a PCO syndrome were evaluated. According to the obtained records 2520 women experienced an improvement of their menstrual cyclicity towards ovulatory cycles. Among them, a total number of 545 women became pregnant. The pregnancies occurred after the intake of two to three months of mayoinositol and folic acid. This means a ratio of 15.1% of the investigated women becomes pregnant during this observational study. No twin pregnancies were documented.

No relevant side effects have been reported in the patients taking myoinositol and folic acid product.

**Figure 2:**
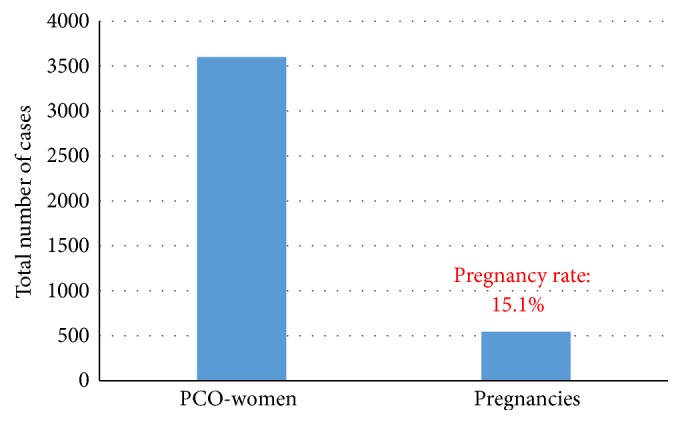
Number of patients and pregnancy rates.

Figure 2 depicts the data. In the subgroup of 32 patients where hormonal values were evaluated a significant improvement of androgen levels and a rise in the progesterone values were observed.

This is shown in Table 1. The Appendix depicts the used German questionnaire. Furthermore, out of these 32 women who became pregnant, 5 of them experienced an abortion, whereas the remaining 27 delivered healthy newborns.

## 4. Discussion

Despite the clear limitations of the observational study, there are reliable available data, since a wide range of patients can be analyzed. This study could show that a new treatment option for patients with a PCOS and infertility is available. Seventy % of the patients restored ovulation after the treatment. Furthermore, the achieved pregnancy rates are at least in a range equivalent to or even superior to those reported by the use of the insulin sensitizer metformin. Karimzadeh and Javedani [12] described a pregnancy rate of 14.4% in a cohort of 90 women and Legro et al. [13] of 12.3% in a cohort of 75 women with PCOS.

The interesting results that the study has shown seem to be related to the mechanism of action of myoinositol. The administration of this molecule, acting as a direct messenger of insulin signaling and improving the glucose tissues uptake, could improve the insulin resistance status of PCOS women, restoring indeed their hormonal status and restoring the ovulation process.

Another important evidence is also related to the difference of myoinositol and metformin in terms of safety profile and compliance for patients. In patients under metformin, side effects have been commonly reported, in particular from mild up to severe gastrointestinal side effects, such as abdominal pain, nausea, and diarrhea. Only in rare cases, very severe side effects as lactic acidosis have been found. On the other side, myoinositol seems to be a safe and well-tolerated approach, anyhow able to give similar results of metformin in terms of clinical efficacy.

In fact, many studies have demonstrated in the last months that an improvement in the rates of ovulation and regularization of menstrual cycles was obtained by the combined use of 4 g myoinositol with 400 *μ*g folic acid per day. Gerli et al. [14] could show in a prospective study that the group of patients receiving myoinositol + folic acid experienced in 82% of the cases an ovulation, whereas this was only observed in 63% of the cases in the group of patients which received a placebo. By the same way 70% of the patients of the myoinositol group developed regular menstrual cycles after 16 weeks of treatment, whereas only 13% of the women did it in the placebo group.

In a study of Raffone et al. [15], where a comparison between the administration of myoinositol (2 × 2000 g + 200 *μ*g per day) and the administration of metformin (1500 mg per day) in women with a PCO syndrome was performed, it could be shown that the number of pregnancies was clearly higher in the myoinositol group than in the metformin group of patients.

Some other studies upon others have shown the efficacy of myoinositol in the improvement of the fertility of PCOS patients due to its improvement of the insulin resistance of these women [16–18].

Many studies have been performed that show that the treatment with myoinositol + folic acid in the classical dosage (2 × 2000 g myoinositol + 200 *μ*g folic acid per day) leads to significant positive changes of metabolic and hormonal parameters. Costantino et al. [19] could show in a double-blinded, placebo controlled study that myoinositol led to a statistically significant improvement of the blood pressure, triglycerides, cholesterol, glucose, and insulin values after a 75 mg oral glucose tolerance test. These improvements were achieved after a treatment period of 16 weeks. The evaluated hormonal values showed a significant decrease of the total and free testosterone serum levels and at the same time the progesterone levels, as a marker of ovulation, experienced a significant rise in the group that received myoinositol (see Table 1). This could show that myoinositol did lead not only to positive changes in metabolic parameters but also to a reduction of elevated androgenic values and subsequently to an improvement of skin problems such as acne or hirsutism.

These data can be supported by our own data as a rise of progesterone from a value of 2.1 ng/mL to a value of 12.3 ng/mL could be observed. By the same time a reduction in the levels of testosterone (from 96.6 ng/mL to 43.3 ng/dL) and free testosterone (from 1.2 ng/mL to 0.35 ng/mL) could also be observed.

A meta-analysis of Unfer et al. [20] could validate these data. This study could also show that, under the investigated studies, where the dosage of 4000 g myoinositol + 400 mg folic acid was used, no side effects were observed, especially those which are seen when other insulin sensitizers like metformin are used in high levels of 1500 mg per day.

Improvement in ovulation induction with myoinositol alone and in combination with clomiphene citrate in polycystic ovarian syndrome in patients with insulin resistance was also confirmed by Kamenov et al. [21]. Whether the addition of melatonin will represent a benefit must be confirmed by more studies but first data suggest this [22].

This confirms that myoinositol is not only an effective alternative in the treatment of PCOS patients but also a secure one as no side effects could be observed in the standard dosage. This is on the other side relevant as the compliance of the use rises resulting in better outcomes in the management of ovulation, hyperandrogenism, and metabolic parameters on patients with a PCOS.

## Figures and Tables

**Table 1:** Hormonal data before and after treatment with myoinositol.

	Total testosterone (ng/dL)	Free testosterone (ng/dL)	Progesterone (ng/mL)
Before treatment	96.6 ± 7.5	1.2 ± 0.7	2.1 ± 0.6
After treatment	43.3 ± 5.3	0.35 ± 0.1	12.3 ± 1.3

## Appendix

### German Questionnaire

*Questionnaire Number*

Birth date of the patient: Month/Year

Intake of Clavella because of Sterility

(1) Sterility: primary - secondary
(2) Since when is sterility known? (Number of months):
(3) Causes for sterility: female - female and male - idiopathic
(4) Functional:
  Cyclical disorders (amenorrhea; oligomenorrhea; anovulation)
  PCOS
  Endometriosis
  Other
(5) Non-functional:
  Tubal disorders
  Immunological disorders
  Adhesions
  Other
(6) Sterility treatment in the medical history
  Clomiphene
  Stimulation with gonadotropins
  IvF/ICSI
  Other
(7) How long treatment with Clavella was performed? Months:
(8) Additional treatment? Yes - No
(9) If yes: which treatment?
(10) Did a pregnancy occur?
(11) If yes; how long after starting treatment?
